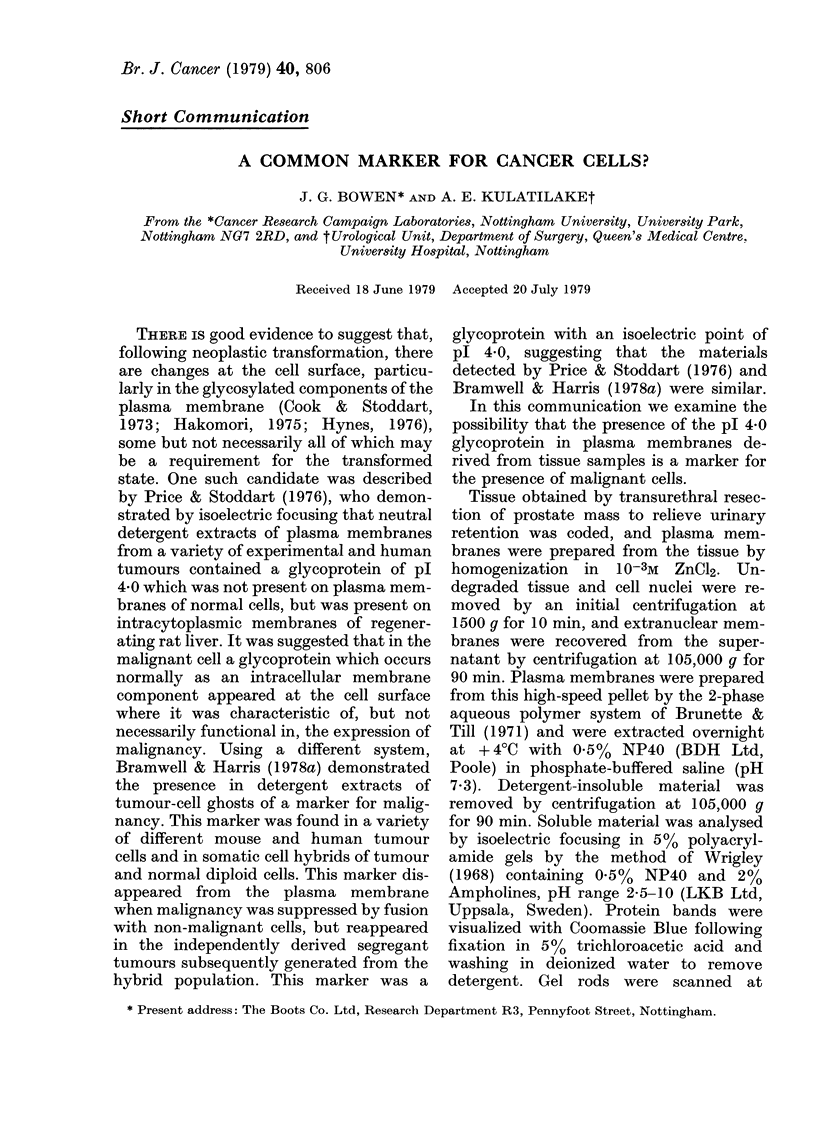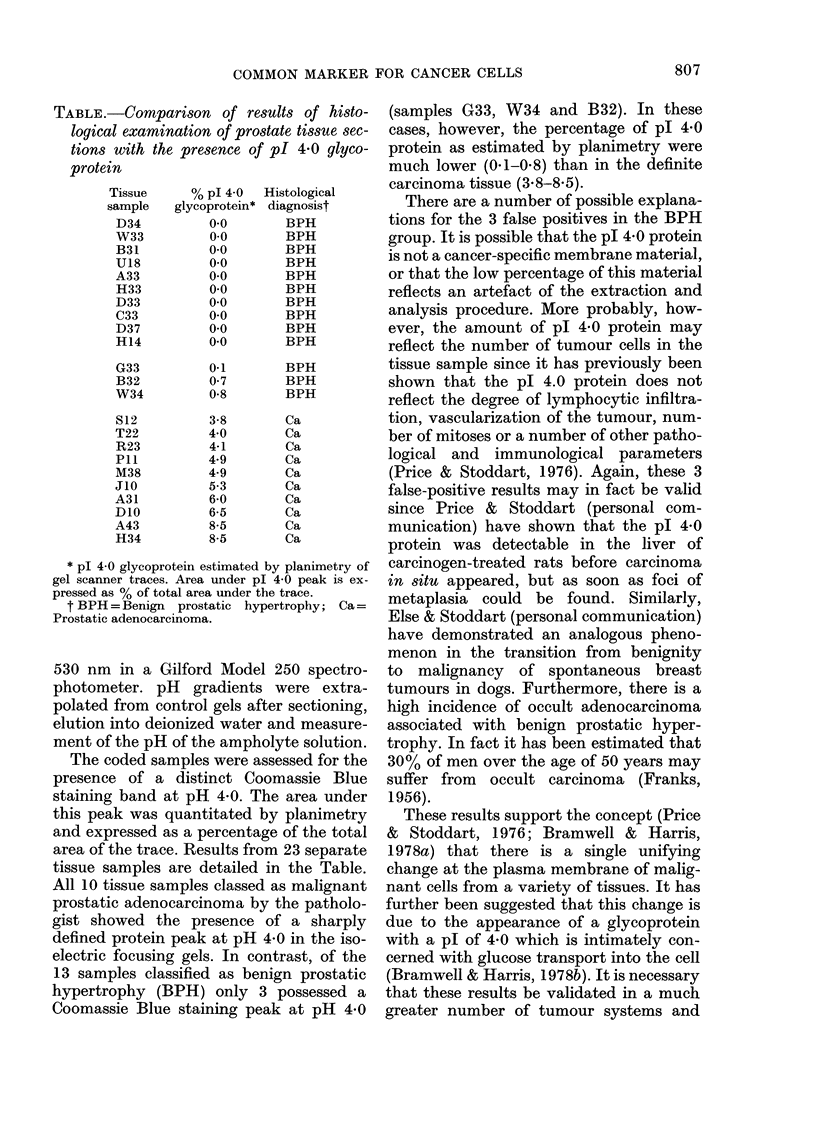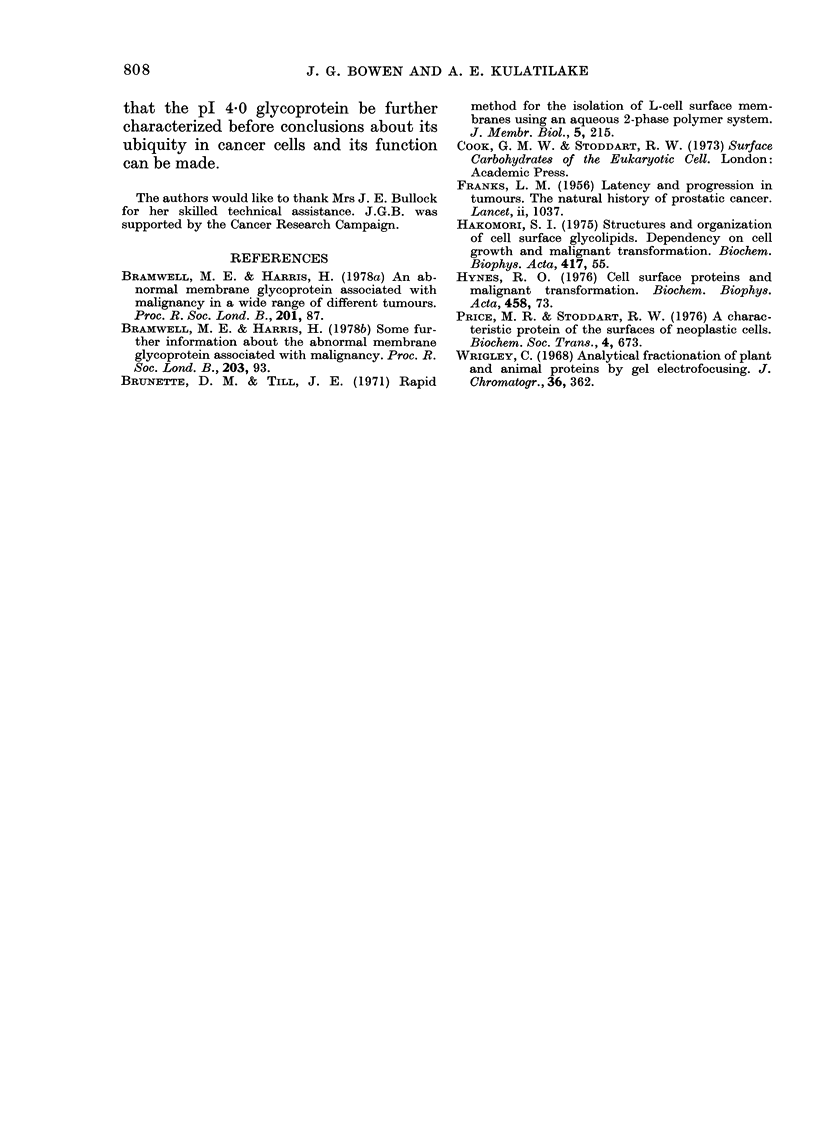# A common marker for cancer cells?

**DOI:** 10.1038/bjc.1979.265

**Published:** 1979-11

**Authors:** J. G. Bowen, A. E. Kulatilake


*Br. J. Cancer* (1979) **40,** 806

***Short Communication***

### A COMMON MARKER FOR CANCER CELLS?

J. G. BOWEN* AND A. E. KULATILAKE†

*From the *Cancer Research Campaign Laboratories, Nottingham University, University Park, Nottingham NG7 2RD, and †Urological Unit, Department of Surgery, Queen's Medical Centre, University Hospital, Nottingham*

Received 18 June 1979 Accepted 20 July 1979

THERE IS good evidence to suggest that, following neoplastic transformation, there are changes at the cell surface, particularly in the glycosylated components of the plasma membrane (Cook & Stoddart, 1973; Hakomori, 1975; Hynes, 1976), some but not necessarily all of which may be a requirement for the transformed state. One such candidate was described by Price & Stoddart (1976), who demonstrated by isoelectric focusing that neutral detergent extracts of plasma membranes from a variety of experimental and human tumours contained a glycoprotein of pI 4·0 which was not present on plasma membranes of normal cells, but was present on intracytoplasmic membranes of regenerating rat liver. It was suggested that in the malignant cell a glycoprotein which occurs normally as an intracellular membrane component appeared at the cell surface where it was characteristic of, but not necessarily functional in, the expression of malignancy. Using a different system, Bramwell & Harris (1978*a*) demonstrated the presence in detergent extracts of tumour-cell ghosts of a marker for malignancy. This marker was found in a variety of different mouse and human tumour cells and in somatic cell hybrids of tumour and normal diploid cells. This marker disappeared from the plasma membrane when malignancy was suppressed by fusion with non-malignant cells, but reappeared in the independently derived segregant tumours subsequently generated from the hybrid population. This marker was a glycoprotein with an isoelectric point of pI 4·0, suggesting that the materials detected by Price & Stoddart (1976) and Bramwell & Harris (1978*a*) were similar.

In this communication we examine the possibility that the presence of the pI 4·0 glycoprotein in plasma membranes derived from tissue samples is a marker for the presence of malignant cells.

Tissue obtained by transurethral resection of prostate mass to relieve urinary retention was coded, and plasma membranes were prepared from the tissue by homogenization in $10^{-3}$M $ZnCl_2$. Undegraded tissue and cell nuclei were removed by an initial centrifugation at 1500 *g* for 10 min, and extranuclear membranes were recovered from the supernatant by centrifugation at 105,000 *g* for 90 min. Plasma membranes were prepared from this high-speed pellet by the 2-phase aqueous polymer system of Brunette & Till (1971) and were extracted overnight at +4°C with 0·5% NP40 (BDH Ltd, Poole) in phosphate-buffered saline (pH 7·3). Detergent-insoluble material was removed by centrifugation at 105,000 *g* for 90 min. Soluble material was analysed by isoelectric focusing in 5% polyacrylamide gels by the method of Wrigley (1968) containing 0·5% NP40 and 2% Ampholines, pH range 2·5–10 (LKB Ltd, Uppsala, Sweden). Protein bands were visualized with Coomassie Blue following fixation in 5% trichloroacetic acid and washing in deionized water to remove detergent. Gel rods were scanned at

* Present address: The Boots Co. Ltd, Research Department R3, Pennyfoot Street, Nottingham.

TABLE.—*Comparison of results of histological examination of prostate tissue sections with the presence of pI 4·0 glycoprotein*

| Tissue sample | % pI 4·0 glycoprotein* | Histological diagnosis† |
|---|---|---|
| D34 | 0·0 | BPH |
| W33 | 0·0 | BPH |
| B31 | 0·0 | BPH |
| U18 | 0·0 | BPH |
| A33 | 0·0 | BPH |
| H33 | 0·0 | BPH |
| D33 | 0·0 | BPH |
| C33 | 0·0 | BPH |
| D37 | 0·0 | BPH |
| H14 | 0·0 | BPH |
| G33 | 0·1 | BPH |
| B32 | 0·7 | BPH |
| W34 | 0·8 | BPH |
| S12 | 3·8 | Ca |
| T22 | 4·0 | Ca |
| R23 | 4·1 | Ca |
| P11 | 4·9 | Ca |
| M38 | 4·9 | Ca |
| J10 | 5·3 | Ca |
| A31 | 6·0 | Ca |
| D10 | 6·5 | Ca |
| A43 | 8·5 | Ca |
| H34 | 8·5 | Ca |

* pI 4·0 glycoprotein estimated by planimetry of gel scanner traces. Area under pI 4·0 peak is expressed as % of total area under the trace.

† BPH = Benign prostatic hypertrophy; Ca = Prostatic adenocarcinoma.

530 nm in a Gilford Model 250 spectrophotometer. pH gradients were extrapolated from control gels after sectioning, elution into deionized water and measurement of the pH of the ampholyte solution.

The coded samples were assessed for the presence of a distinct Coomassie Blue staining band at pH 4·0. The area under this peak was quantitated by planimetry and expressed as a percentage of the total area of the trace. Results from 23 separate tissue samples are detailed in the Table. All 10 tissue samples classed as malignant prostatic adenocarcinoma by the pathologist showed the presence of a sharply defined protein peak at pH 4·0 in the isoelectric focusing gels. In contrast, of the 13 samples classified as benign prostatic hypertrophy (BPH) only 3 possessed a Coomassie Blue staining peak at pH 4·0 (samples G33, W34 and B32). In these cases, however, the percentage of pI 4·0 protein as estimated by planimetry were much lower (0·1–0·8) than in the definite carcinoma tissue (3·8–8·5).

There are a number of possible explanations for the 3 false positives in the BPH group. It is possible that the pI 4·0 protein is not a cancer-specific membrane material, or that the low percentage of this material reflects an artefact of the extraction and analysis procedure. More probably, however, the amount of pI 4·0 protein may reflect the number of tumour cells in the tissue sample since it has previously been shown that the pI 4.0 protein does not reflect the degree of lymphocytic infiltration, vascularization of the tumour, number of mitoses or a number of other pathological and immunological parameters (Price & Stoddart, 1976). Again, these 3 false-positive results may in fact be valid since Price & Stoddart (personal communication) have shown that the pI 4·0 protein was detectable in the liver of carcinogen-treated rats before carcinoma *in situ* appeared, but as soon as foci of metaplasia could be found. Similarly, Else & Stoddart (personal communication) have demonstrated an analogous phenomenon in the transition from benignity to malignancy of spontaneous breast tumours in dogs. Furthermore, there is a high incidence of occult adenocarcinoma associated with benign prostatic hypertrophy. In fact it has been estimated that 30% of men over the age of 50 years may suffer from occult carcinoma (Franks, 1956).

These results support the concept (Price & Stoddart, 1976; Bramwell & Harris, 1978*a*) that there is a single unifying change at the plasma membrane of malignant cells from a variety of tissues. It has further been suggested that this change is due to the appearance of a glycoprotein with a pI of 4·0 which is intimately concerned with glucose transport into the cell (Bramwell & Harris, 1978*b*). It is necessary that these results be validated in a much greater number of tumour systems and that the pI 4·0 glycoprotein be further characterized before conclusions about its ubiquity in cancer cells and its function can be made.

The authors would like to thank Mrs J. E. Bullock for her skilled technical assistance. J.G.B. was supported by the Cancer Research Campaign.

### REFERENCES

BRAMWELL, M. E. & HARRIS, H. (1978*a*) An abnormal membrane glycoprotein associated with malignancy in a wide range of different tumours. *Proc. R. Soc. Lond. B.*, **201**, 87.

BRAMWELL, M. E. & HARRIS, H. (1978*b*) Some further information about the abnormal membrane glycoprotein associated with malignancy. *Proc. R. Soc. Lond. B.*, **203**, 93.

BRUNETTE, D. M. & TILL, J. E. (1971) Rapid method for the isolation of L-cell surface membranes using an aqueous 2-phase polymer system. *J. Membr. Biol.*, **5**, 215.

COOK, G. M. W. & STODDART, R. W. (1973) *Surface Carbohydrates of the Eukaryotic Cell.* London: Academic Press.

FRANKS, L. M. (1956) Latency and progression in tumours. The natural history of prostatic cancer. *Lancet*, ii, 1037.

HAKOMORI, S. I. (1975) Structures and organization of cell surface glycolipids. Dependency on cell growth and malignant transformation. *Biochem. Biophys. Acta*, **417**, 55.

HYNES, R. O. (1976) Cell surface proteins and malignant transformation. *Biochem. Biophys. Acta*, **458**, 73.

PRICE, M. R. & STODDART, R. W. (1976) A characteristic protein of the surfaces of neoplastic cells. *Biochem. Soc. Trans.*, **4**, 673.

WRIGLEY, C. (1968) Analytical fractionation of plant and animal proteins by gel electrofocusing. *J. Chromatogr.*, **36**, 362.